# The phenomenon of spontaneous tumor regression in breast cancer

**DOI:** 10.1093/jscr/rjad651

**Published:** 2023-12-05

**Authors:** Abid Qureshi, Sindhuri Gollamudi, Shahar Qureshi, Nisha Sondhi, Shahzaib Nabi, Romulo Genato, Philip Xiao, Armand Asarian

**Affiliations:** Department of Surgery, The Brooklyn Hospital Center, Brooklyn, NY 11201, United States; Department of Internal Medicine, The Brooklyn Hospital Center, Brooklyn, NY 11201, United States; Department of Surgery, The Brooklyn Hospital Center, Brooklyn, NY 11201, United States; Department of Surgery, The Brooklyn Hospital Center, Brooklyn, NY 11201, United States; Department of Oncology, The Brooklyn Hospital Center, Brooklyn, NY 11201, United States; Department of Surgery, The Brooklyn Hospital Center, Icahn School of Medicine at Mount Sinai, Brooklyn, NY 11201, United States; Department of Pathology, The Brooklyn Hospital Center, Icahn School of Medicine at Mount Sinai, Brooklyn, NY 11201, United States; Department of Surgery, The Brooklyn Hospital Center, Icahn School of Medicine at Mount Sinai, Brooklyn, NY 11201, United States

**Keywords:** spontaneous, regression, tumor, cancer, malignancy, breast

## Abstract

Spontaneous tumor regression is an increasingly prevalent phenomenon of partial or complete disappearance of primary tumor tissue or associated metastases in the absence of therapeutic intervention. Cases of spontaneous regression have been established in malignant tumors, such as testicular germ cell tumor, renal cell cancer, melanoma, basal cell carcinoma, neuroblastoma, colon cancer, breast cancer, as well as metastases. Breast cancer has increasingly been reported to have a higher rate of spontaneous regression than previously thought. Immunologic response is cited as the forefront of spontaneous regression phenomenon, with the focus on immunologic cell death. This report brings awareness to a case of spontaneous regression observed in invasive ductal carcinoma of the breast and how disruption of the tumor microenvironment can take a variable course even in malignant disease.

## Introduction

Spontaneous tumor regression is an increasingly prevalent phenomenon of partial or complete disappearance of primary tumor tissue or associated metastases in the absence of therapeutic intervention, with frequency approximately ranging in 1 in every 60 000–100 000 cancer cases. Cases of spontaneous regression have been established in malignant tumors, such as testicular germ cell tumor, renal cell cancer, melanoma, basal cell carcinoma, neuroblastoma, colon cancer, breast cancer, as well as metastases [1]. Specifically, regression of breast cancers has previously been denoted in various studies including a literature review highlighting spontaneous regression from 1900 to 1987 of which 43/741 cases were breast cancer [2]. Many hypotheses working in simultaneous progression have been cited to explain the phenomenon. Activation of the host’s immunological response and oncotic apoptotic processes has so far been the main driving pathological mechanisms [3]. This report brings awareness to a case of spontaneous regression observed in invasive ductal carcinoma of the breast and how disruption of the tumor microenvironment can take a variable course even in malignant disease.

## Case report

**Figure 1:**
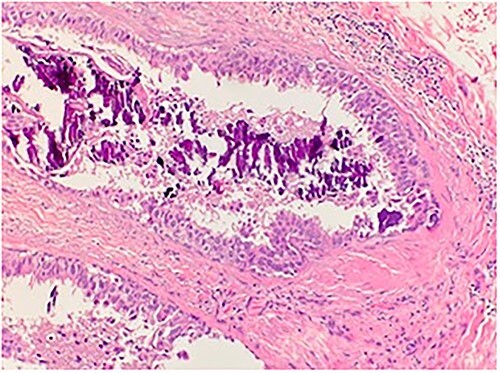
Microscopic examination shows high grade ductal carcinoma cells confined within the base membrane with necrosis. H&E 20×.

**Figure 2:**
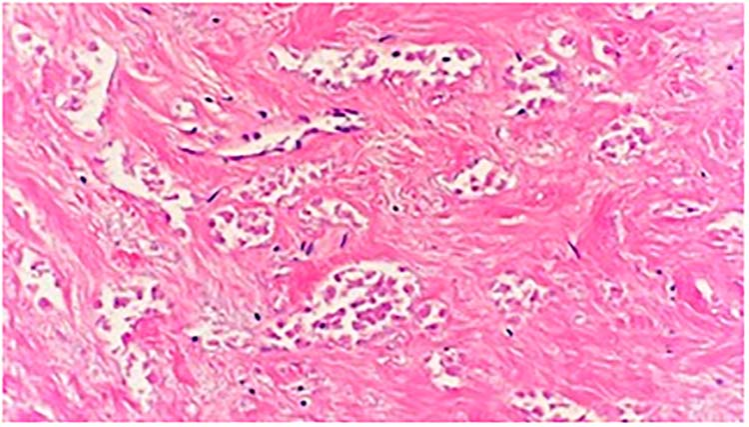
Microscopic examination shows a sheet of nonviable tumor cells with necrosis. H&E 20×.

An 84-year-old female presented to the breast surgery office for biopsy proven malignant 2-cm palpable mass on physical examination in the right retroareolar region. Her medical history was significant for hypertension, diabetes mellitus, and stroke. She had a diagnostic bilateral mammogram and sonogram along with a biopsy of the site in question, at another outside facility, which had yielded invasive ductal carcinoma with perineural invasion. In addition, indeterminate linear microcalcifications were visualized in the central right breast 3-cm posterior to the mass and also in the central far posterior lower right breast. These warranted another diagnostic mammogram and sonogram. Repeat diagnostic mammogram and sonogram were remarkable for: biopsy-proven malignant 1.2-cm mass in the right retroareolar region. Additional suspicious microcalcifications 3- and 7-cm posterior to the mass were noted, classified as BIRADS 4. She underwent a stereotactic biopsy. Pathology for the retroareolar mass was positive for moderately differentiated invasive ductal carcinoma with perineural invasion and pathology for calcifications was positive for Ductal Carcinoma in Situ (Fig. 1). Invasive ductal carcinoma was positive for ER (95%), PR (50%), and negative for Her-2/Neu. Ductal Carcinoma In Situ was positive for ER (90%–95%) and negative for PR (0%). Subsequently, upon an extensive discussion with the various options including but not limited to lumpectomy and mastectomy, she elected to undergo a right simple mastectomy without reconstruction with right axillary sentinel node biopsy. Surgical pathology was remarkable for no evidence of active malignancy in any of the previously biopsied areas; evidence of nodular necrosis of tumor cells noted (Fig. 2) consistent with inflammatory reaction and fibrocystic changes. Patient was started on Anastrozole and the following postoperative course was unremarkable.

## Discussion

The phenomenon of spontaneous tumor regression of cancer is one of the most fascinating occurrences in medicine. Observations of it have been dated back to at least hundreds of years. Cole and Everson [4] published the accepted criteria for the phenomenon as the “partial or complete disappearance of a malignant tumor in the absence of all treatment, or in the presence of therapy which is considered inadequate to exert a significant influence on neoplastic disease” under the criteria that cancer was originally proven microscopically present. Out of the 47 with breast cancer in the same study, four were observed to have undergone spontaneous regression (8.5%). They further went on to cite some possible explanations for the phenomenon including: endocrine influences with effectiveness of either endocrine therapy in cancer or hormonal changes within the body, surgical removal indicating the possibility of remaining tissue being inflammatory, and others such as unusual sensitivity to radiotherapy, infection, allergic reaction, or interference of blood supply of the tumor during tumor biopsy/incomplete removal [4]. Since then, many other mechanistic interpretations, including tumor necrosis and trauma, have been added.

Malignancy has previously demonstrated the capability to impede the immune system via inhibition of cytotoxic/signaling effects, bypassing and restricting antigen recognition causing tumor evasion, and lymphocytic depletion. Tumor also variably invokes the nutrient supply, including amino acids and glucose, of immune cells by the accumulation of specific signaling molecules and metabolites. Another focus of cancer evasion is the alteration of lymphocyte metabolism, growth, and maturation through the inhibition of growth factors, receptors, and ligands [5]. All of these factors foreplay into a rich microenvironment of the tumor, leading to progressive growth of cancer.

In the 1990s, Papac [6] explored the different concepts surrounding spontaneous regression and initially reviewed data suggesting the involvement of cancer apoptosis and differentiation of malignant to benign tumor. Later, he reported that the mechanisms were multifocal immunologic theories surrounding immune mediation, hormonal mediation, tumor necrosis, tumor and angiogenesis inhibition via cytokines or growth factors, apoptotic and epigenetic mechanisms [7]. It is clear that various immunologic mechanisms simultaneously work together to either differentiate tumors or cause regression by cell death.

Immunologic response is cited as the forefront of spontaneous regression phenomenon, with the focus on immunologic cell death (ICD). ICD is a form of cell death that involves the host innate immune system and its use of immune memory including adaptive immunity that can help create advantageous systemic effects with several effects at play. Innate immunity is the first response the body induces against threat and incorporates lymphocytes, natural killer (NK) cells or other immune cells, as well as antibodies. Under the influence of interleukin signaling molecules and without prior sensitization, NK cells and T lymphocytes are directly cytotoxic to tumors. Tumor cells are also recognized by the immune system by expression of Class 1 human leukocyte antigen on cell membrane, with upregulation of cytotoxic T cells, resulting in tumor cell death [2, 8]. Adaptive immunity is based on tumor associated antigen processing via antigen processing cells, such as dendritic cells (DCs) and their association with long-term immunity. For example, chronic exposure of damage-associated molecular patterns acts as a stimulatory signal to attract immune cells like DCs and their activation into a mature phenotype, promoting the engulfment of antigenic components. Consequently, via antigen presentation, DCs stimulate specific T-cell responses to kill cancer cells. The induction of ICD eventually results in long-lasting protective autoimmunity and immune-related disruption of tumor microenvironment [8].

Our report highlights a case of spontaneous regression that was noted in a patient with biopsy proven invasive ductal carcinoma of the breast and DCIS in two separate areas of the same breast. Given there was no active malignancy remaining on pathology specimens without any treatment, the case fits the ideology of spontaneous regression phenomenon. In this case, it is possible the biopsies disrupted the tumor microenvironment and alerted the immune response to impede further tumor progression as well as kill cancer cells. In a prior study, tumor microenvironment manipulation by surgical invasion, either by excision or biopsy, has been cited as a possibility of increasing host immune system response of ICD and natural defense against tumors [9]. Our case distinctly indicates that spontaneous regression of breast cancer is associated with ICD.

The concept of spontaneous regression is increasingly being studied and reported in literature. Regression is more commonly associated with groups of tumors like the embryonal tumors in children, carcinoma of the female breast, chorionepithelioma, adenocarcinoma of the kidney, neuroblastoma, malignant melanoma, sarcomas, and carcinoma of the bladder and skin [10]. Breast cancer has increasingly been reported to have a higher rate of spontaneous regression than previously thought. Although because of novel diagnostic and therapeutic treatments, occurrences of spontaneous regression may be decreasing as the process of natural host defense and immunity to appropriately take effect is cut short by the treatment [11]. Despite this, it is difficult to predict which breast cancers will undergo spontaneous regression versus requirement for therapy. It is still best to follow the standard breast cancer therapeutic guidelines. Despite certain limitations, there is hope that understanding the mechanisms behind spontaneous regression could lead to replication of the process in a more targeted and refined way of treating cancer overall including development of immunotherapies and progression preventative strategies.
